# The Epidermal Growth Factor Receptor (EGFR / HER-1) Gatekeeper Mutation T790M Is Present in European Patients with Early Breast Cancer

**DOI:** 10.1371/journal.pone.0134398

**Published:** 2015-08-12

**Authors:** Vahid Bemanian, Torill Sauer, Joel Touma, Bjørn Arne Lindstedt, Ying Chen, Hilde Presterud Ødegård, Katja Marjaana Vetvik, Ida Rashida Bukholm, Jürgen Geisler

**Affiliations:** 1 Department of Gene Technology, Akershus University Hospital, Lørenskog, Norway; 2 University of Oslo, Institute of Clinical Medicine, Campus at Akershus University Hospital, Lørenskog, Norway; 3 Department of Pathology, Akershus University Hospital, Lørenskog, Norway; 4 Department of Breast- and Endocrine Surgery, Akershus University Hospital, Lørenskog, Norway; 5 Department of Oncology, Akershus University Hospital, Lørenskog, Norway; Seoul National University, REPUBLIC OF KOREA

## Abstract

The epidermal growth factor receptor (EGFR) is one of the major oncogenes identified in a variety of human malignancies including breast cancer (BC). EGFR-mutations have been studied in lung cancer for some years and are established as important markers in guiding therapy with tyrosine kinase inhibitors (TKIs). In contrast, EGFR-mutations have been reported to be rare if not absent in human BC, although recent evidence has suggested a significant worldwide variation in somatic EGFR-mutations. Therefore, we investigated the presence of EGFR-mutations in 131 norwegian patients diagnosed with early breast cancer using real-time PCR methods. In the present study we identified three patients with an EGFR-T790M-mutation. The PCR-findings were confirmed by direct Sanger sequencing. Two patients had triple-negative BC (TNBC) while the third was classified as luminal-A subtype. The difference in incidence of T790M mutations comparing the TNBC subgroup with the other BC subgroups was statistical significant (*P* = 0.023). No other EGFR mutations were identified in the entire cohort. Interestingly, none of the patients had received any previous cancer treatment. To our best knowledge, the EGFR-T790M-TKI-resistance mutation has not been previously detected in breast cancer patients. Our findings contrast with the observations made in lung cancer patients where the EGFR-T790M-mutation is classified as a typical „second mutation”causing resistance to TKI-therapy during ongoing anticancer therapy. In conclusion, we have demonstrated for the first time that the EGFR-T790M-mutation occurs in primary human breast cancer patients. In the present study the EGFR-T790M mutation was not accompanied by any simultaneous EGFR-activating mutation.

## Introduction

The epidermal growth factor receptor (EGFR / HER-1) is one of the major oncogenes identified in a variety of human cancers including breast cancer [1–5]. Genes functioning in the epidermal growth factor signalling pathway are among the most frequently activated oncogenes in human cancers [6, 7]. While EGFR overexpression and / or amplification have been shown to occur frequently in human breast cancer [8–10], EGFR mutations are thought to be rare if not absent [11–18].

However, an increasing body of evidence suggests significant worldwide variation in somatic EGFR mutations in breast cancer patients [19, 20]. To our knowledge, the EGFR mutational status has not been investigated in breast cancer patients from Norway. Therefore, the aim of the present study was to examine the presence of relevant somatic EGFR mutations in Norwegian breast cancer patients. We intended to include all typical subgroups of breast cancer patients representing the major entities, including luminal-A, luminal-B, HER-2-positive, and triple-negative/basal-like-type breast cancers. The triple-negative patients were of particular interest as these have received much attention in the research community due to their severe prognosis and the lack of clinically usefull biomarkers that may guide therapy [21–23].

EGFR-mutational analysis from asian groups have explored the presence of EGFR mutations in breast cancer patients [13], however there are limited data regarding caucasian cohorts. As EGFR has been identified as a promising target for cancer patients for some time, several potent drugs, (e.g. Gefitinib, Erlotinib, Cetuximab, Lapatinib etc.), all approved for the treatment of cancer patients, have been tested in clinical breast cancer studies with overall disappointing results [4]. Thus, tyrosine kinase inhibitors like gefitinib and erlotinib did not significantly improve response rates in early clinical studies involving breast cancer patients [24–26]. Possible explanations for the observed lack of efficacy in these trials may be poor patient selection criteria and enrollment of heavily pretreated patients in these early trials. Recently improved understanding of the role of EGFR in breast cancer biology has highlighted that new clinical trials involving EGFR-inhibitors aimed at highly selected patient populations may we warranted.

## Patients and Methods

Patients diagnosed with early breast cancer were asked to contribute to a research biobank located at the Akershus University Hospital (University of Oslo, Campus AHUS, Norway). 168 unselected (consecutive) patients aged 36–91 years were chosen for the analysis. Due to lack of sufficient tumor material some patients (n = 36) had to be excluded from the analysis. In addition, one patient was registered with duplicate samples, leaving 131 cases for the final assessment. All patients were diagnosed with early breast cancer suitable for immediate surgery in the time period 2007–2008. After surgery, all patients received standard adjuvant treatment according to the national treatment guidelines published by the Norwegian Breast Cancer Group (NBCG; www.nbcg.net) in collaboration with the Norwegian Health Authorities. No experimental therapy was given at any time as part of this study. All patients gave written informed consent prior to participation. This study and biobank were approved by the Regional Committee for Medical and Health Research Ethic (REC) REC SOUTH EAST NORWAY (postal address: Postbox 1130, Blindern, 0318 Oslo, Norway; approval number: 2014-895-REC SOUTH EAST).

### Tissue samples

**Fig 1:**
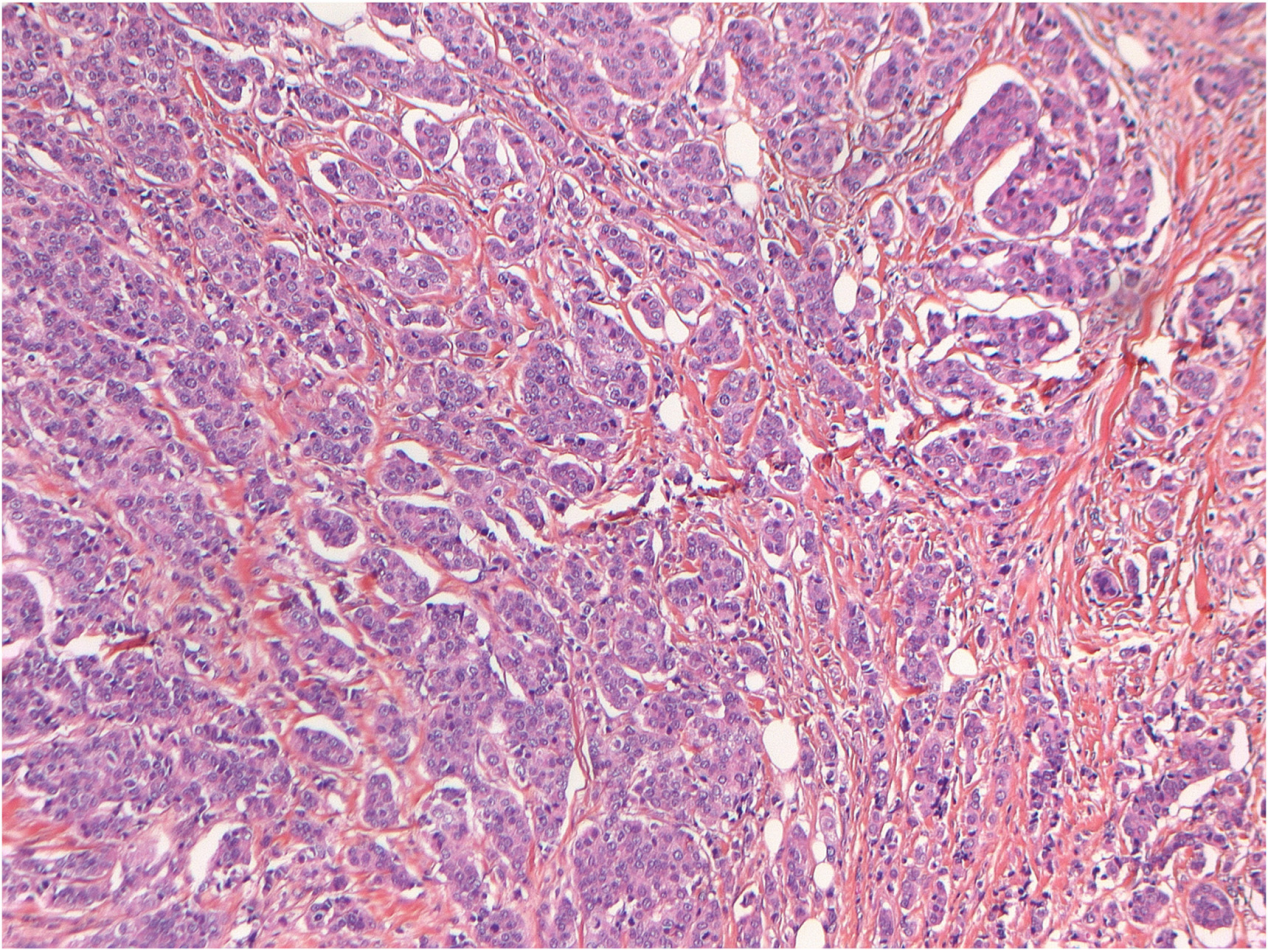
Quality control of specimens. Typical section of an infiltrating ductal carcinoma (IDC), grade III, showing high tumor cell content of the sample prior to analysis.

Tumor samples were obtained during breast surgery in addition to routine diagnostic biopsies (formalin-fixated, paraffin-embedded biopsies; FFPE). The standard diagnostic dataset, used to determine the need of adjuvant therapies i.e. type of breast cancer, grading, ER-status, PGR-status, and HER-2 status were obtained from the diagnostic biopsies. All research biopsies were evaluated by a pathologist specialised in breast cancer diagnostics prior to analysis to ensure that the biopsies contained adequate tumor tissue for analysis. A typical example of a control-slide, verifying a high content of tumor cells, is shown (Fig 1).

### Preparation of genomic DNA

Genomic DNA was extracted from formalin-fixed, paraffin-embedded tumor tissue using 4 sections of 10 μm for each sample. Briefly, the paraffin was removed from sample using deparaffinization solution (Qiagen). Tumor tissue was lysed using proteinase K digestion incubated overnight at 56°C. Genomic DNA was then purified from the lysate using the QIAsymphony DSP DNA Mini kits (Qiagen) and Qiagen´s fully automated QIAsymphony SP/AS robot. The final concentration of genomic DNA in each sample was measured on a NanoDrop 2000 spectrophotometer.

### EGFR mutation analysis

A panel of EGFR-mutations was detected in DNA samples from 131 individuals using the Therascreen EGFR RGQ PCR kit (Qiagen Inc.) according to the manufacturer´s protocol. The samples were analysed for point mutations in exon 18 (G719A/C/S), in-frame deletions in exon 19, in-frame insertions as well as point mutations in exon 20 (S768I, T790M) and point mutations in exon 21 (L858R and L861Q). For the EGFR mutation analysis the extracted DNA was diluted to 5 ng/μl and 25 ng DNA was used for each PCR reaction. In addition to the controls included in the kits, a sample with a known EGFR-mutation was used as a kit-independent control. The mutations found by PCR were confirmed subsequently by Sanger sequencing using gene-specific primers as described below.

### Direct Sanger sequence analysis of samples with EGFR-mutations

The mutation status of the EGFR T790M-positive samples was subsequently confirmed by automated Sanger dideoxy sequencing. Briefly, 100 ng genomic DNA was amplified using Platinum Taq DNA polymerase (Life Technologies) and gene-specific primers (Table 1). The PCR cycling conditions were as follows: initial denaturation 95°C/2 minutes; 42 amplification cycles (denaturation 95°C/20 seconds; annealing 55°C/30 seconds; synthesis 72°C/30 seconds) followed by a final synthesis at 72°C for 5 minutes. The PCR product was gel-purified using QIAquick gel extraction kit (Qiagen). The purified PCR products were analysed by direct automatic PCR-assisted sequencing using Big Dye terminator V1.1 (Life Technologies) according to the supplier´s protocol. The PCR cycling conditions were as follows: 25 cycles 96°C/10 seconds; 50°C/5 seconds; 60°C/4 minutes. The PCR products were purified using BigDye Xterminator purification kit (Life Technologies). Analysis of the purified sequenced products was performed on a 3130XL Genetic Analyzer (Life Technologies). The DNA sequences were analyzed by Sequencher DNA Sequence Analysis software (Genecodes).

**Table 1:** Primers used for direct Sanger-sequencing of EGFR-Exon 20

EGFR-Exon 20-F	5´-GCA-TCT-GCC-TCA-CCT-CCA-C-3
EGFR-Exon 20-R	5´-CTG-GCT-CCT-TAT-CTC-CCC-TC-3
EGFR-Exon 20-R2	5´-GTC-TTT-GTG-TTC-CCG-GAC-AT-3

### Statistical analysis

Descriptive and comparative statistical analysis was performed using the IBM SPSS program (Version 22). Mutation frequencies were compared across three clinical breast cancer subsets including: (1) Luminal-A/B, (2) HER-2 positive BC, and (3) triple-negative BC using the Fisher´s exact test. P-values < 0.05 were considered significant. All P-values are given as "mid-p corrected" values to compensate for multiple testing etc.

## Results

**Fig 2:**
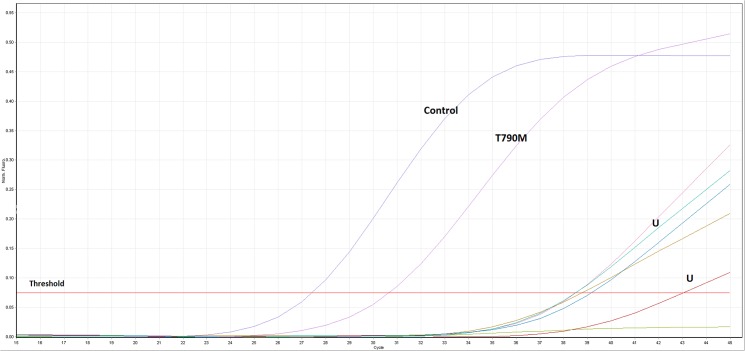
Real-time PCR analysis. Real-time PCR analysis of the EGFR-T790M point mutation. The figure shows a representative sample showing the T790M point mutation detected in a patient sample: the control-curve indicates the amplification of a region of exon 2 of the EGFR gene and is used to assess the total DNA in the sample while the T790M-curve indicates the amplification of the T790M-mutant allele in the sample. The other curves labelled with U indicate unspecific amplification of DNA (not present in further analysis).

**Fig 3:**
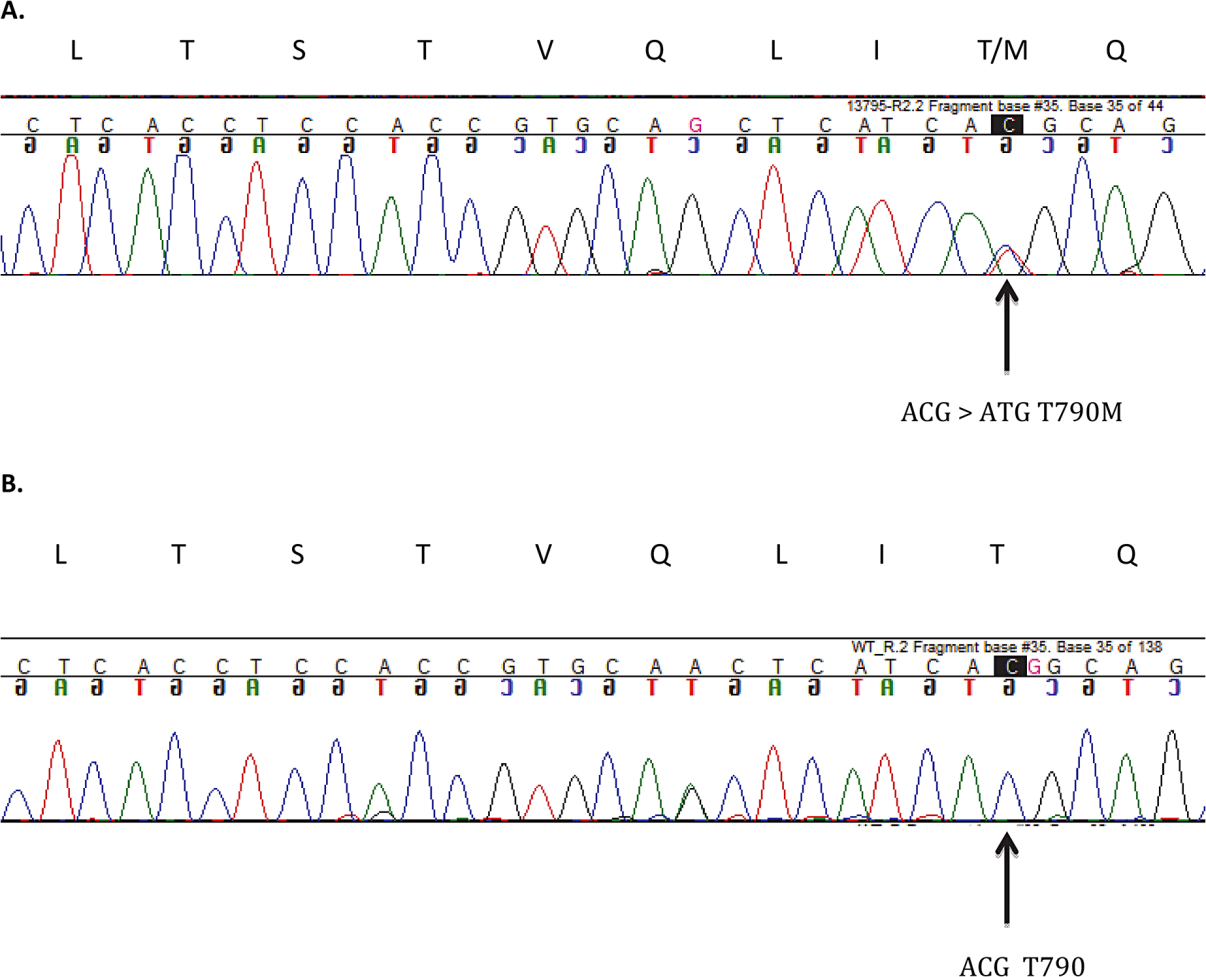
Sanger sequencing. Sanger sequencing of the EGFR point mutation in a positive breast cancer sample (A) compared to wild-type control (B). The peak of the mutated allele (ACG > ATG) is indicated by arrow. The upper panel indicates parts of the amino acid sequence encoded by EGFR exon 20.

One hundred and thirty one individual breast cancer cases were included in this study. Table 2 summarizes the standard clinical and pathology data (immunhistochemistry etc.). Tumors were classified as luminal-A/B (n = 82), HER-2 positive (n = 32; either HER-2 IHC 3+ or amplified, irrespective of ER/PGR-status) or triple-negative (n = 17). Using tumor biopsies obtained from these 131 individual patients with early breast cancer, we identified all in all three patients with EGFR mutations. The tumor classification, staging and immunhistochemistry (ER, PGR, HER-2, grading etc.) for these three patients is summarized in Table 3. Notably, staining for Ki-67 was not considered a standard procedure in Norway in 2007/2008 and is therefore not available. Thus, the luminal-A and luminal-B patients are reported as one group. In all three BC cases with EGFR-mutations, a point mutation was identified situated in exon 20 of EGFR, a threonine-to-methionine mutation at codon 790 (T790M). The real-time-PCR curves for one of the three patients with an EGFR T790M mutation is shown in Fig 2. Two of the three patients had triple-negative breast cancer, while the third one had a classical luminal-A-profile (Table 3). Therefore, while the T790M-mutation was present in 11.8% of all triple-negative BC patients involved in this study (2 out of 17), it was only identified in 1.2% (1 out of 82) of patients with luminal-A/B BC. The incidence of EGFR T790M mutations in the TNBC subgroup was statistically significant higher compared to the other BC subgroups (*P* = 0.023). The PCR findings were subsequently confirmed by traditional Sanger sequencing (Fig 3). All three T790M mutations were somatic mutations and present in approximately 25–50% of the DNA. No other EGFR-mutations were identified in the study cohort. None of the three EGFR-T790M-positive patients had a known immigration background.

**Table 2:** Patients´ characteristics: frequencies and percentage (n = 131).

		(n)	(%)
**Histology**	IDC	102	77.9
	ILC	16	12.2
	MED	1	0.8
	Others	12	9.2
**Grading**	Grade I	8	6.1
	Grade II	69	52.7
	Grade III	54	41.2
**TNM**	T1	61	46.6
	T2	64	48.9
	T3	6	4.6
	N0	74	56.5
	N1	33	25.2
	N2	13	9.9
	N3	11	8.4
	M0	129	98.5
	M1	2	1.5
**ER**	pos.	97	74
	neg.	34	26
**PGR**	pos.	60	45.8
	neg.	71	54.2
**HER-2**	pos.	32	24.4
	neg.	96	73.3
**BC-subtypes**	LUM-A/B	82	62.6
	HER-2 pos.	32	24.4
	TNBC	17	13.0

IDC, infiltrating ductal carcinoma; ILC, infiltrating lobular carcinoma; MED, medullary carcinoma; ER, estrogen receptor; PGR, progesterone receptor; HER-2, human epidermal growth factor receptor type II; BC, breast cancer; LUM-A, luminal A subtype; LUM-B, luminal B subtype; HER-2 pos., Human Epidermal growth factor Receptor type II positive (confirmed either by immunohistochemistry 3+ or by in-situ-hybridisation techniques); TNBC, triple-negative breast cancer

**Table 3:** Patients´characteristics: three individual Norwegian breast cancer patients with EGFR T790M mutations.

Pat.-no.	Age*	Histo.	grade	T	N	M	ER	PGR	HER-2	Subtype
1	87	IDC	III	2	3	0	pos.	neg.	neg.	LUM.-A
					(25 of 25)		(100%)	(<10%)	(IHC: 0)	
2	42	IDC	III	2	0	0	neg.	neg.	neg.	TNBC
					(0 of 1, SNP)	0	(0%)	(0%)	(IHC: 0)	
3	72	IDC	III	2	0	0	neg.	neg.	neg.	TNBC
		(PMCA)			(0 of 13)		(0%)	(0%)	(IHC: 0)	

*Age, age at breast cancer surgery; IDC, infiltrating ductal carcinoma; IHC, immunohistochemistry; PMCA, pleomorph carcinoma (subtype of IDC); LUM-A, luminal A subtype; SNP, sentinel node procedure; TNBC, triple-negative breast cancer subtype

## Discussion

The presence of EGFR-activating mutations is established as one of the most important predictive markers for targeted anticancer therapy [5]. While considerable groundbreaking cancer research related to EGFR and its mutations is based on research in lung cancer patients [4, 27], breast cancer is also known to be strongly associated with EGFR stimulated cell growth [28–31]. Early clinical studies testing the efficacy of EGFR-inhibitors in metastatic breast cancer delivered mostly disappointing results and resistance to EGFR-targeting drugs emerged as a major barrier to their clinical use [24–26]. The mechanisms of resistance to EGFR targeted therapies have been discussed extensively in the literature and are not discussed here [32].

**Fig 4:**
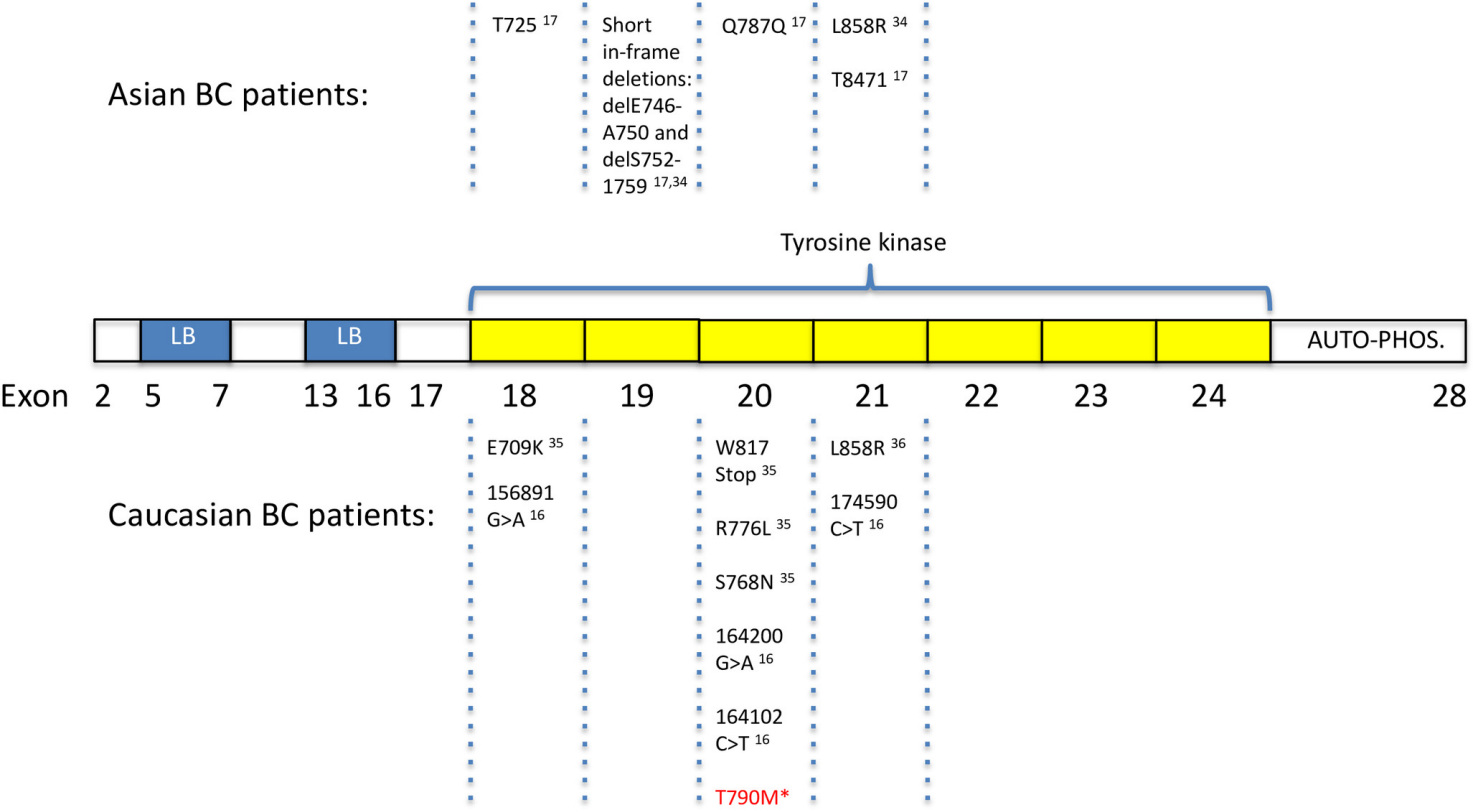
Overview: Typical EGFR mutations and polymorphisms identified hitherto in sporadic breast cancer. LB, ligand binding; Auto-phos., autophosphorylation; *present publication.

Due to the general importance of the EGFR-pathway in breast cancer biology, the identification of EGFR-mutations in breast cancer may aid further investigation as well as guide the use of EGFR inhibitors [33]. Several investigators have studied the EGFR-mutational status in breast cancer patients (as summarized in Fig 4) from different global regions with variable results [19, 20]. Using a patient cohort from Singapore, Teng et al. [17] reported 8 activating EGFR mutations in exons 19 and 21 in 70 breast cancer samples (11.4%). These findings, supporting a role for EGFR mutations in a subset of BC patients were recently strengthened by a publication of Lv et al. identifying two EGFR activating mutations in 139 chinese patients [34]. EGFR missense mutations in sporadic and hereditary breast cancer have been published by Weber et al. [35] suggesting that the spectra of somatic EGFR mutations is higher in the tumor stroma compared to the neoplastic epithelium. In addition, it was suggested by the same authors that missense mutations occur more frequently in BRCA1/2-positive tumors compared to sporadic BC. Santarpia et al. reported the activating mutation EGFR L858 (c.2573T>G) to be present in 2.6% of patients recruited mainly at the University of Texas M.D. Anderson Cancer Center (MDACC, Houston, TX) [36]. However, not all reports have identified EGFR mutations in BC cohorts. Jacot et al. [19] studied 229 TNBC patients from Europe and did not report any activating EGFR mutations. In addition, at least three other european research groups have published the absence of EGFR activating mutations in their cohorts of breast cancer patients [14–16].

Due to these invariable results, our aim was to evaluate Norwegian patients for the existence of activating EGFR mutations presenting with and being treated for breast cancer in a Norwegian University Hospital. Much to our surprise, EGFR T790M mutations were identified in three BC patients. No other simultaneous EGFR mutations were detected by the screening methodology employed. The patients from our institution were all operable at diagnosis and have never received any kind of anticancer therapies including TKIs prior to surgery and biobanking. EGFR T790M mutant tumors are characterized by dramatically increased ATP affinity explaining the clinically observed drug resistance during therapy with ATP-competitive kinase inhibitors [37]. Our results contrast the findings made in lung cancer patients where the EGFR T790M mutation is a typical „second mutation”observed under therapeutic pressure during ongoing TKI-therapy [37, 38]. Interestingly, two of our three patients had triple-negative breast cancer (TNBC) lacking useful predictive markers guiding anticancer therapy. The third patient belonged to the luminal-A subtype.

Several authors have suggested anti-EGFR treatment to be ineffective due to the largely negative results obtained during phase II studies performed with gefitinib in breast cancer cohorts [24–26]. In addition, it has been shown that the number of BC patients with EGFR mutations is very low in general. Thus, unselected BC patients will in general not benefit from these treatment strategies. However, positive trial results from lung cancer patients with EGFR activating mutations treated with EGFR inhibitors suggest it may be worthwhile to identify BC patients with similar EGFR mutations and treat them in modern studies aiming at precision medicine. With novel technological opportunities allowing broader testing for somatic mutations in human tumor biopsies at lower costs, such a strategy appears to be increasingly applicable. In addition, our finding that two out of three T790M mutated tumors were found in TNBC, might suggest that EGFR-mutations may play a role in subgroups of TNBC patients, lacking otherwise predictive markers. While our finding of isolated T790M mutations is intriguing, the biological impact on intracellular signalling and tumor growth in general is currently unknown. Indeed, all three EGFR T790M positive BC patients in the present study lack the typically accomodating activating mutations in EGFR.

To the best of our knowledge, we have identified for the first time, the EGFR T790M „TKI-resistance”mutation in a subgroup of early breast cancer patients in Northern Europe. We suggest that BC patients, in particular TNBC patients, should be tested for activating and other EGFR mutations at a larger scale allowing the treatment of suitable patients with novel targeted therapies in clinical trials involving for example novel „pan-HER-inhibitors”affecting also T790M-mutated EGFR [39]. The biological impact of an isolated EGFR T790M mutation in BC patients should be further investigated to determine its role as a breast cancer driver and its potential as a target for therapeutic intervention.
